# Identification and comparative analysis of the *CIPK* gene family and characterization of the cold stress response in the woody plant *Prunus mume*

**DOI:** 10.7717/peerj.6847

**Published:** 2019-04-30

**Authors:** Ping Li, Tangchun Zheng, Lulu Li, Xiaokang Zhuo, Liangbao Jiang, Jia Wang, Tangren Cheng, Qixiang Zhang

**Affiliations:** 1 Beijing Key Laboratory of Ornamental Plants Germplasm Innovation & Molecular Breeding, Beijing Forestry University, Beijing, China; 2 National Engineering Research Center for Floriculture, Beijing Forestry University, Beijing, China; 3 Beijing Laboratory of Urban and Rural Ecological Environment, Beijing Forestry University, Beijing, China; 4 Engineering Research Center of Landscape Environment of Ministry of Education, Beijing Forestry University, Beijing, China; 5 Key Laboratory of Genetics and Breeding in Forest Trees and Ornamental Plants of Ministry of Education, Beijing Forestry University, Beijing, China; 6 Beijing Advanced Innovation Center for Tree Breeding by Molecular Design, Beijing Forestry University, Beijing, China

**Keywords:** *CIPK* gene family, Genetic evolution, Expression pattern, *Prunus mume*, Cold response

## Abstract

*Prunus mume* is an important ornamental woody plant that grows in tropical and subtropical regions. Freezing stress can adversely impact plant productivity and limit the expansion of geographical locations. Understanding cold-responsive genes could potentially bring about the development of new ways to enhance plant freezing tolerance. Members of the serine/threonine protein kinase (*CIPK*) gene family play important roles in abiotic stress. However, the function of *CIPK* genes in *P. mume* remains poorly defined. A total of 16 *CIPK* genes were first identified in *P. mume*. A systematic phylogenetic analysis was conducted in which 253 *CIPK* genes from 12 species were divided into three groups. Furthermore, we analysed the chromosomal locations, molecular structures, motifs and domains of *CIPK* genes in *P. mume*. All of the CIPK sequences had NAF domains and promoter regions containing cis-acting regulatory elements of the related stress response. Three *PmCIPK* genes were identified as Pmu-miR172/167-targeted sites. Transcriptome data showed that most *PmCIPK* genes presented tissue-specific and time-specific expression profiles. Nine genes were highly expressed in flower buds in December and January, and 12 genes were up-regulated in stems in winter. The expression levels of 12 *PmCIPK* genes were up-regulated during cold stress treatment confirmed by qRT-PCR. Our study improves understanding of the role of the *PmCIPK* gene family in the low temperature response in woody plants and provides key candidate genes and a theoretical basis for cold resistance molecular-assisted breeding technology in *P. mume*.

## Introduction

Low temperature damage is an environmental stress that severely limits the geographic distribution and cultivation range of perennial plants (Weiser, 1970). Plants have evolved specific and effective molecular mechanisms to defend against low temperature injury. A number of functional genes of the cold response have been confirmed in plants, and some of these genes are closely related to Ca^2+^ (e.g., C-repeat binding factor, CBF). Ca^2+^ signals represent a universal transduction signal in plants that is translated by elaborate Ca^2+^-binding proteins, many of which function as Ca^2+^ sensors and act on downstream responses (Kudla et al., 2018). The large number of probable SCaBP/CBL-PKS/CIPK combinations indicate that the Ca^2+^/SOS3/SOS2 signalling pathway is widely used in plants (Zhu, 2001, 2002). Calcineurin B-like proteins (CBLs) form functional complexes with CBL-interacting protein kinases (CIPKs, SnRK3s) to relay plant responses to many environmental signals and to regulate ion fluxes (Hashimoto et al., 2012), and the CBL-CIPK complexes perform important functions in the signal transduction pathways in which Ca^2+^ is a second messenger, especially for various non-biological signals that regulate ion transporter activity (Luan, 2009; Zhu, 2016). The function of the CBL-CIPK network has been investigated quite intensively in recent years. In *Populus euphratica*, PeCBL/PeCIPK complexes have been identified and shown to be functional in the regulation of Na^+^/K^+^ homeostasis (Zhang et al., 2013a).

During the last few decades, many CBL-CIPK complexes have been shown to be involved in signal transduction during responses to salt and osmotic stress conditions; however, few studies have concentrated on the role of the CBL-CIPK network during the cold stress response in plants. Recent studies have revealed that the *CIPK* gene family showed significant increases in transcript after cold stress treatments (Chen et al., 2011; Niu et al., 2018). The plasma membrane protein COLD1 senses cold stress and produces a cytosolic Ca^2+^ signal. Calcium-dependent protein kinases (CPKs) and CBL-CIPK complexes transmit Ca^2+^ signalling to activate the mitogen-activated protein (MAP) kinase cascade, and activated MAPKs induce the phosphorylation of transcription factors (TFs) such as calmodulin-binding transcription activators and inducer of CBF expressions (ICEs) genes to induce the expression of cold-responsive genes (Zhu, 2016). Expression patterns indicated that *ZmCIPK* genes were up-regulated under abiotic stress, and 19 *ZmCIPK* genes responded to cold stress (Chen et al., 2011). The protein kinase CIPK7 is activated by the CBL1 to enhance cold tolerance (Huang et al., 2011). The transcript levels of 4 *BnaCIPK* genes showed significant increases after cold stress treatment (Zhang et al., 2014). Overexpression of *OsCIPK03* increased the tolerance of positive transgenic plantlets to cold stress (Xiang, Huang & Xiong, 2007).

CIPKs exhibit a conserved modular structure comprising a CIPK-specific C-terminal regulatory domain and a junction domain (Stout, Foster & Matthews, 2004). The latter contains the phosphatase interaction domain (PPI) and the autoregulatory NAF domain (Albrecht et al., 2001). The NAF domain, as the minimum protein module required for interaction, is both essential and sufficient to mediate interaction with the CBL calcium sensor proteins (Leulliot et al., 2007). The NAF domain, a 24 amino acid domain named after the characteristic amino acids N, A, and F, is found in a plant-specific subgroup of CIPKs that interact with CBLs (Albrecht et al., 2001). Upon the interaction of CBLs with CIPKs, the auto-inhibitory NAF domain is released from the protein domain, producing an active kinase conformation (Weinl & Kudla, 2009). Whereas, the N-terminal part of CIPKs includes a conserved catalytic domain typical of serine/threonine kinases, the much less conserved C-terminal domain is unique to serine/threonine protein kinases (Stout, Foster & Matthews, 2004).

*Prunus mume* is an important ornamental woody plant with diverse features that incorporates winter flowering, colorful petals, a characteristic aroma, and green branches (Zhang et al., 2018a). *P. mume* also exhibits early flowering and can enrich the landscaping of cold areas in early spring. As a woody plant native to southern China, *P. mume*, which tolerates temperatures as low as −19 °C in the dormant period, has been domesticated for a long time, and partial species have been cultivated in East Asia. However, *P. mume* is more sensitive to low temperatures than other woody plants such as *Acer negundo* and *Viburnum plicatum* var. tomentosum (Irving & Lanphear, 1967). Therefore, low temperature plays a key limiting factor for *P. mume* survival and growth in regions of low temperature. Previous studies of *CIPK* genes have focused on herbaceous plants, no report of *CIPK* genes in woody plants. Recently, whole genome sequencing and genome resequencing of *P. mume* were completed, laying a foundation for exploring the molecular mechanism of cold resistance in *P. mume* at the molecular level (Zhang et al., 2012, 2018a). Our aims are to clarify whether *PmCIPK* genes respond to low temperatures in *P. mume* and provide new insight into the further molecular dissection of biological functions for cold tolerance in perennial woody plants.

## Materials and Methods

### Identification of *CIPK* Genes in *P. mume* and other species

Based on the CIPK protein sequences reviewed (SWISS-PROT, https://www.uniprot.org/) (The UniProt Consortium, 2015), including those from *A. thaliana* and *O. sativa*, a CIPK model was built using HMMScan software (Finn et al., 2015). The CIPK model along with the protein kinase (PF00069) and NAF domains (PF03822), which were downloaded from the Pfam database (https://pfam.xfam.org/) (Finn et al., 2008), were used as queries to search the genome sequences of *P. mume* (Zhang et al., 2012)*, Prunus persica* (The International Peach Genome Initiative et al., 2013), *Fragaria vesca* (Shulaev et al., 2011), *Rosa chinensis* (Raymond et al., 2018), *Prunus avium* (Shirasawa et al., 2017), *Malus × domestica* (Velasco et al., 2010)*, Pyrus bretschneideri* (Wu et al., 2013), *Pyrus communis* (Chagné et al., 2014), *Rubus occidentalis* (Chagné et al., 2014), and *Prunus yedoensis* (VanBuren et al., 2016). The *P. mume* genome was obtained from the *P. mume* genome project, while other Rosaceae genomes were downloaded from the Phytozome (https://phytozome.jgi.doe.gov/pz/portal.html#) and GDR databases (https://www.rosaceae.org/). Then, a HMMER search (*E*-value < 1e-6) was employed to identify the *CIPK* gene family members. Based on positional information in the *P. mume* genome project, the physical chromosomal locations of *PmCIPK* genes were plotted using MapChart software (Voorrips, 2002).

### Phylogenetic tree construction and calculation of *Ka*/*Ks* ratios

To study the phylogenetic relationships between *CIPK* genes in *P. mume* and other species, CIPK proteins from three species (*P. mume*, *A. thaliana*, and *O. sativa*) and CIPK proteins from nine Rosaceae species were used in a multiple sequence alignment with ClustalX 2.0.11 software (Larkin et al., 2007). Subsequently, a phylogenetic tree based on the sequences was constructed via the maximum likelihood (ML) method using 1,000 replicate bootstrap values and the Jones-Taylor-Thornton model using MEGA X (Kumar et al., 2018). Phylogenetic trees from CIPK sequences were annotated and embellished using the online Evolview tool (http://www.evolgenius.info/evolview/#login) (He et al., 2016).

Duplicate PmCIPK sequences were found using a plant genome duplication database (http://chibba.agtec.uga.edu/duplication/) (Lee et al., 2013). The nonsynonymous (*Ka*) and synonymous (*Ks*) substitution of *PmCIPK* sequences were estimated using DnaSP software (Lee et al., 2013) to predict the divergence time (*t*) and evolutionary strain (*Ka*/*Ks* ratio). Based on two common rates (λ) of 1.5 × 10^−8^ or 6.1 × 10^−9^ substitutions per site per year (Blanc & Wolfe, 2004; Lynch & Conery, 2000), the divergence time was calculated using the formula *t* = *Ks*/2λ × 10^−6^ Mya.

### Gene structures, protein tertiary structures and motif prediction

The exon/intron structures of *PmCIPK* genes were obtained with Gene Structure Display Server 2.0 (Hu et al., 2015) using genomic sequences and structural information. The *Pm*CIPK protein sequences were submitted for multiple expectation maximization for motif elicitation (http://meme-suite.org/index.html) (Brown et al., 2013) analysis to identify conserved motifs and structural divergence. The PmCIPK proteins were submitted to Pfam and SMART (http://smart.embl-heidelberg.de/) for analysis and confirmation of the CIPK-specific functional domains. The tertiary structures and homologs of PmCIPKs were predicted using the online server Phyre2 (http://www.sbg.bio.ic.ac.uk/phyre2/html) (Kelley & Sternberg, 2009).

### Promoter cis-element and miR167/miR172 target site analysis

A total of 1.5 kb-upstream sequences of each *PmCIPK* gene were predicted using the PlantCARE database (http://bioinformatics.psb.ugent.be/webtools/plantcare/html/) (Lescot et al., 2002). All of the *PmCIPK* gene family members were predicted for Pmu-miR167 and Pmu-miR172 by psRNATarget (http://plantgrn.noble.org/psRNATarget/) (Dai, Zhuang & Zhao, 2018). The Mfold web server (http://unafold.rna.albany.edu/) (Bindewald, Kluth & Shapiro, 2010) was used to predict miRNA secondary structure.

### Expression analysis of *PmCIPK* genes

To investigate the *PmCIPK* expression patterns involved in tissue development and cold stress tolerance, raw data from the RNA sequencing of various tissues (i.e., bud, leaf, root, stem, fruit, and flower buds sampled in November, December, January, and March) in *P. mume* were obtained (Zhang et al., 2018b). Transcriptome data from three periods (autumn, October; winter, January; and spring, March) and three different places (Beijing (BJ, N39°54′, E116°28′), Gongzhuling (GZL, N43°42′, E124°47′), and Chifeng (CF, N42°17′, E118°58′)) were analysed under low temperature growth conditions. To compare the gene expression patterns of *CIPK* genes across *P. mume*, *A. thaliana,* and *O. sative* during cold stress, the publicly-available RNA-seq data were downloaded from the National Center for Biotechnology Information GEO DataSets (GSE112225 and GSE67373). The HeatMapper online tool (Babicki et al., 2016) was used to generate the heat map. Molecular interactions of PmCIPKs were analysed using KEGG PATHWAY (https://www.kegg.jp/kegg/pathway.html) (Kanehisa et al., 2017) and STRING (https://string-db.org/) (Szklarczyk et al., 2015).

### Plant material and qRT-PCR analysis

A total of 6-month-old seedlings at 24 °C under long-day conditions (16-h light/8-h dark) were used for examining the effect of *PmCIPK* genes on the cold response. We incubated seedlings in soil at 4 °C at approximately 65% humidity. Leaves from treated seedlings were sampled at 0, 1, 4, 6, 12, and 24 h for total RNA isolation. First strand cDNA synthesis was performed using a TIANScript First Strand cDNA Synthesis Kit (Tiangen, Beijing, China) according to the manufacturer’s instructions. qRT-PCR was carried out using a PikoReal real-time PCR system (Thermo Fisher Scientific, CA, USA) with SYBR® Premix Ex*Taq* TM (TaKaRa, Dalian, China). The reactions were performed in a 10 μL volume containing 5 μL of SYBR® Premix Ex *Taq* II, 0.25 μL each of forward and reverse primers (Table S1), 0.5 μL of cDNA and 3 μL of ddH_2_O. The reactions were completed with the following conditions: 95 °C for 30 s, 40 cycles of 95 °C for 5 s and 60 °C for 40 s, 60 °C for 30 s, and an end step at 20 °C. The analyses were confirmed in triplicate. The relative expression levels of *PmCIPK* genes were calculated using the 2^−ΔΔCt^ method with the *protein phosphatase 2A* gene from *P. mume* as the reference gene. The final data were subjected to an analysis of variance test.

## Results

### Identification of *CIPK* genes in *P. mume*

Based on the HMMER search using the CIPK model, 16 non-redundant PmCIPKs were identified in the *P. mume* genome, and 194 CIPKs were identified in the other 10 species from the Rosaceae genome. The putative CIPKs were named based on the hmmsearch *E*-value of NAF domain (Table S2). Among PmCIPK proteins, there were sequences with a high level of similarity (i.e., more than 51% identical on average). The predicted sizes of the 16 PmCIPKs ranged from 420 (PmCIPK15) to 508 (PmCIPK16) amino acids (aa), and average molecular weight (MW) was 51.24 kDa. The predicted isoelectric points (*pI*) varied from 7.01 (PmCIPK15) to 9.59 (PmCIPK3), and all of these proteins were alkaline (*pI* > 7) (Table 1). *PmCIPK* genes were unevenly distributed in the *P. mume* genome. The *PmCIPK* genes were located in all of the chromosomes and were densely distributed on Chr1, Chr2, and Chr3, which contained five, five and three genes, respectively. However, Chr4, Chr6, and Chr7 contained no *PmCIPK* genes (Fig. S1). A similar phenomenon of unbalanced chromosomal distribution of *CIPK* genes was also shown in the apple (Niu et al., 2018). The unbalanced distribution of genes may be related to species evolution and genetic variation.

**Table 1:** The *PmCIPK* gene family members in *P. mume*.

Name	Gene ID	CDS (bp)	Locus	Exons	Length (aa)	MW (Da)	*pI*
*PmCIPK1*	Pm010243	1,458	Pa3:3391662..3393587	1	486	54,490.0	7.52
*PmCIPK2*	Pm000275	1,392	Pa1:1685196..1687810	2	464	52,896.8	8.8
*PmCIPK3*	Pm019034	1,323	Pa5:21808882..21811580	3	441	49,728.7	9.59
*PmCIPK4*	Pm003063	1,524	Pa1:22944723..22946626	2	421	47,779.5	7.23
*PmCIPK5*	Pm001690	1,317	Pa1:12516375..12523774	14	439	50,327.2	7.14
*PmCIPK6*	Pm018300	1,341	Pa5:17446293..17450538	13	447	51,306.0	8.92
*PmCIPK7*	Pm010242	1,392	Pa3:3385471..3388752	1	464	52,117.5	9.02
*PmCIPK8*	Pm010458	1,374	Pa3:4546104..4551920	15	458	51,489.1	9.5
*PmCIPK9*	Pm027719	1,296	Pa8:16682154..16684142	1	432	48,696.7	9.16
*PmCIPK10*	Pm005699	1,347	Pa2:12440265..12442240	1	449	50,504.3	8.43
*PmCIPK11*	Pm000271	1,392	Pa1:1667938..1670150	1	464	51,942.4	8.42
*PmCIPK12*	Pm008685	1,407	Pa2:35109225..35112580	15	469	52,688.0	7.94
*PmCIPK13*	Pm008498	1,377	Pa2:33557522..33562215	12	459	51,410.7	7.16
*PmCIPK14*	Pm004339	1,347	Pa2:4162663..4167479	14	449	51,103.8	7.68
*PmCIPK15*	Pm009181	1,260	Pa2:39252837..39254427	1	420	45,743.3	7.01
*PmCIPK16*	Pm003066	1,263	Pa1:22959631..22962640	3	508	57,584.1	7.91

### Phylogenetic tree analysis and calculation of *Ka*/*Ks* ratios

**Figure 1:**
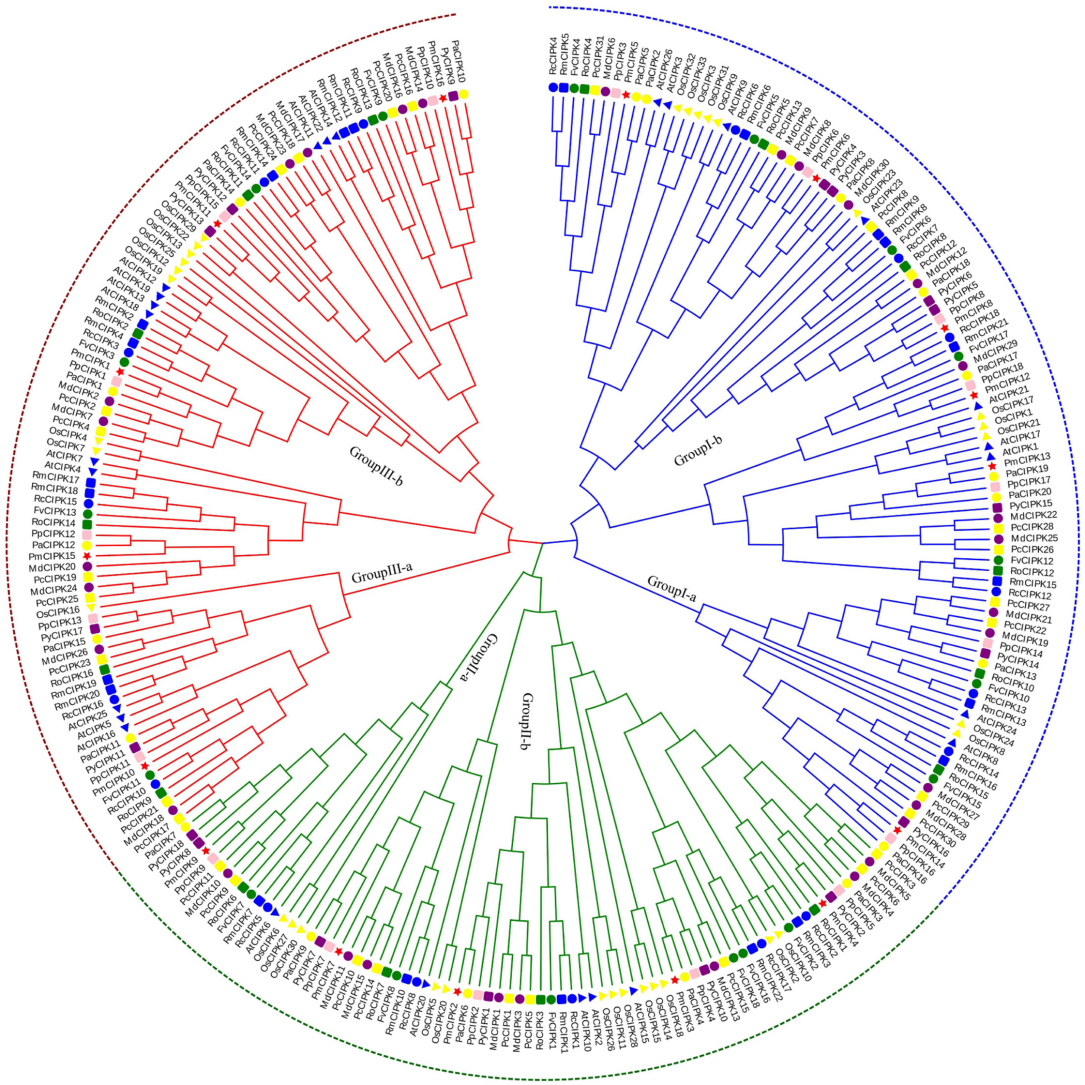
Phylogenetic tree of CIPK sequences from *P. mume* and other plant species. The Group I, Group II, and Group III subfamilies are indicated by blue, red, and green branch lines, respectively. Pm, *P. mume*; At, *A. thaliana*; Os, *O. sativa*; Pp, *P. persica*; Fv, *F. vesca*; Rc, *R. chinensis*; Pa, *P. avium*; Md, *M. × domestica*; Pb, *P. bretschneideri*; Pc, *P. communis*; Ro, *R. occidentalis*; Py, *P. yedoensis var. nudiflora*. Different species are labeled with different colors and shape display markers while PmCIPKs are labeled with red star display markers.

According to multiple sequence alignments, we used the ML method to construct a phylogenetic tree of all CIPK sequences from *P. mume*, *A. thaliana*, and *O. sativa.* Based on the reviewed (SWISS-PROT) AtCIPKs and OsCIPKs, 16 PmCIPKs were divided into three groups (i.e., Group I, Group II, and Group III) (Fig. S2). To better understand the evolutionary relationships between CIPKs, a ML phylogenetic tree was constructed using the CIPKs from 12 species, including 10 Rosaceae species (Fig. 1). Similar to the tree structure described above, the CIPKs were divided into three groups, and every group included similar clades (I-a, I-b; II-a, II-b; and III-a, III-b). Group I was the largest group, containing 100 CIPKs, whereas group III was the smallest group, consisting of 77 members, indicating that CIPKs were distributed unevenly in the different groups. The CIPKs from the Rosaceae genus were distributed uniformly in every small clade, whereas CIPKs from *O. sativa* tended to cluster together. Notably, all intron-rich PmCIPKs were clustered in Group I, similar to the clustering of CIPKs from *Zea mays* (Chen et al., 2011). The PmCIPKs, PpCIPKs, and PaCIPKs were clustered together and had similar distributions in the phylogenetic tree.

In the present study, three *PmCIPK* gene pairs were identified (*PmCIPK11/3*, *PmCIPK16/3*, and *PmCIPK10/9*), indicating that whole genome duplication/segmental duplications (SDs) may have been involved in the expansion of the *CIPK* gene family in *P. mume* (Table S3). During their evolution, a *Ka*/*Ks* ratio > 1 implies positive selection (adaptive evolution), a ratio = 1 implies neutral evolution (drift), and a ratio <1 implies negative selection (conservation). The *Ka*/*Ks* ratio of only one *PmCIPK* gene pair (*PmCIPK10/9*), which was 0.28, has meaning and shows there has been synonymous change. The divergence period of the *PmCIPK10/9* gene pair ranged from 41.24 to 76.38 Mya.

### Motif prediction, gene structure, and protein tertiary structures

**Figure 2:**
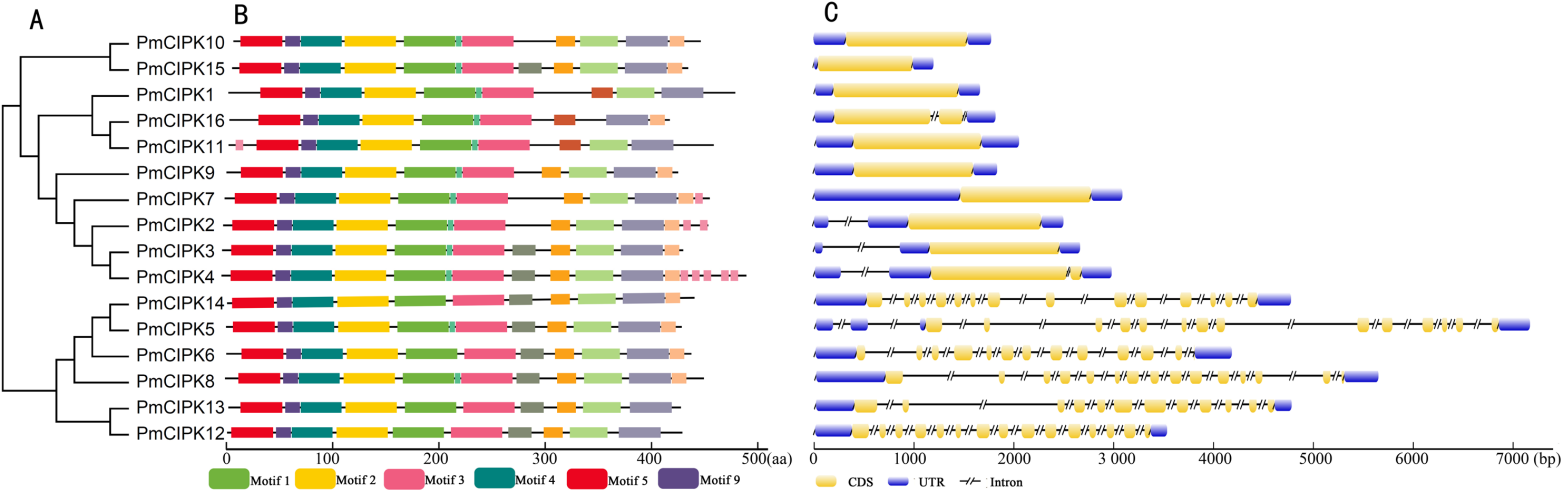
Phylogenetic analysis and gene structure of *PmCIPK* genes. (A) Phylogenetic tree was constructed based on the full amino acid sequences of PmCIPKs. (B) Motif distribution of PmCIPK proteins. Protein motif architectures were indicated using Pfam and MEME online tool. (C) Exons and introns structures of *PmCIPK* genes. The yellow round-corner rectangle represents exons, the black shrinked line represents introns, and the blue round-corner rectangle represents UTR.

The serine/threonine protein kinases are likely found in all eukaryotes and have roles in a multitude of cellular processes. The kinase, NAF, and PPI domains are the basic characteristics of the CIPK family in plants. The conserved domain showed that all 16 PmCIPK contained the kinase, NAF, and PPI domains (Fig. 2B). The results of MEME analysis showed that all PmCIPK proteins shared six motifs that were contained within the typical domains of plant CIPKs (protein kinase domain and NAF domain). A total of 14 distinct motifs were identified, and motif 1, motif 2, motif 3, motif 4, motif 5, and motif 9 were found in all of the PmCIPKs (Fig. S3). Furthermore, most of the PmCIPKs contained similar motifs, and these motifs are in similar places with similar sizes in the PmCIPK sequences. Similar permutations of motifs exhibited close phylogenetic relationships between the *PmCIPK* genes (Fig. 2).

The exon-intron diversity is not only an important part of gene family evolution but also may influence their expression. To understand the annotated features of the *PmCIPK* genes, we used GSDS 2.0 to construct the coordinates of the exon-intron. The results showed that out of 16 *PmCIPKs*, 10 genes (62.5%) comprised the largest/smallest numbers of exons, which were mainly concentrated in the range of one to three, while six genes (37.5%) contained multiple exons. We found that closely clustered *PmCIPK* genes in the same clades have similar exon number, UTR exon number, and even the number of exons with significant differences in the *PmCIPK* genes. Interestingly, the evolutionary relationships of the *PmCIPK*s were divided into two groups based on different numbers of exons (Fig. 2C).

The three-dimensional structures of 16 PmCIPK proteins were predicted (Fig. S4). Using a template model, all CIPK sequences were modelled with 100.0% confidence by the single highest scoring template. Five identical top-scoring proteins for all PmCIPK sequences were found. Hypothetical protein c6c9dB belonged to the serine/threonine-protein kinase MARK1, while the other four belonged to the protein kinase family. Serine/threonine-protein kinase MARK1 (c6c9dB) shared 28–45% sequence identity with the PmCIPKs, which was anticipated as it is a homologue of CIPK, with 100% probability. On the other hand, the other four protein kinases (c5ebzF, c4wnkA, c3pfqA, and c4cfh) shared 24–44% sequence identity with the PmCIPKs (Table 2; Table S4).

**Table 2:** The confidence and sequence identities of the homologous relationships of the PmCIPKs.

Name	c6c9dB_		c5ebzF_		c4wnkA_		c3pfqA_		c4cfhA_	
	%confidence	%identity	%confidence	%identity	%confidence	%identity	%confidence	%identity	%confidence	%identity
PmCIPK1	100	43	100	30	100	29	100	32	100	42
PmCIPK2	100	28	100	25	100	29	100	31	100	30
PmCIPK3	100	32	100	27	100	27	100	33	100	31
PmCIPK4	100	30	100	29	100	25	100	28	100	30
PmCIPK5	100	31	100	27	100	28	100	29	100	27
PmCIPK6	100	30	100	31	100	27	100	31	100	36
PmCIPK7	100	29	100	28	100	24	100	30	100	30
PmCIPK8	100	30	100	30	100	28	100	28	100	34
PmCIPK9	100	29	100	27	100	26	100	31	100	30
PmCIPK10	100	30	100	28	100	29	100	32	100	30
PmCIPK11	100	45	100	29	100	26	100	29	100	41
PmCIPK12	100	42	100	29	100	25	100	28	100	44
PmCIPK13	100	32	100	27	100	30	100	34	100	34
PmCIPK14	100	30	100	30	100	30	100	30	100	34
PmCIPK15	100	39	100	26	100	24	100	27	100	34
PmCIPK16	100	29	100	28	100	24	100	27	100	38

### Cis-acting regulatory elements and miR167/miR172 target site analysis

**Figure 3:**
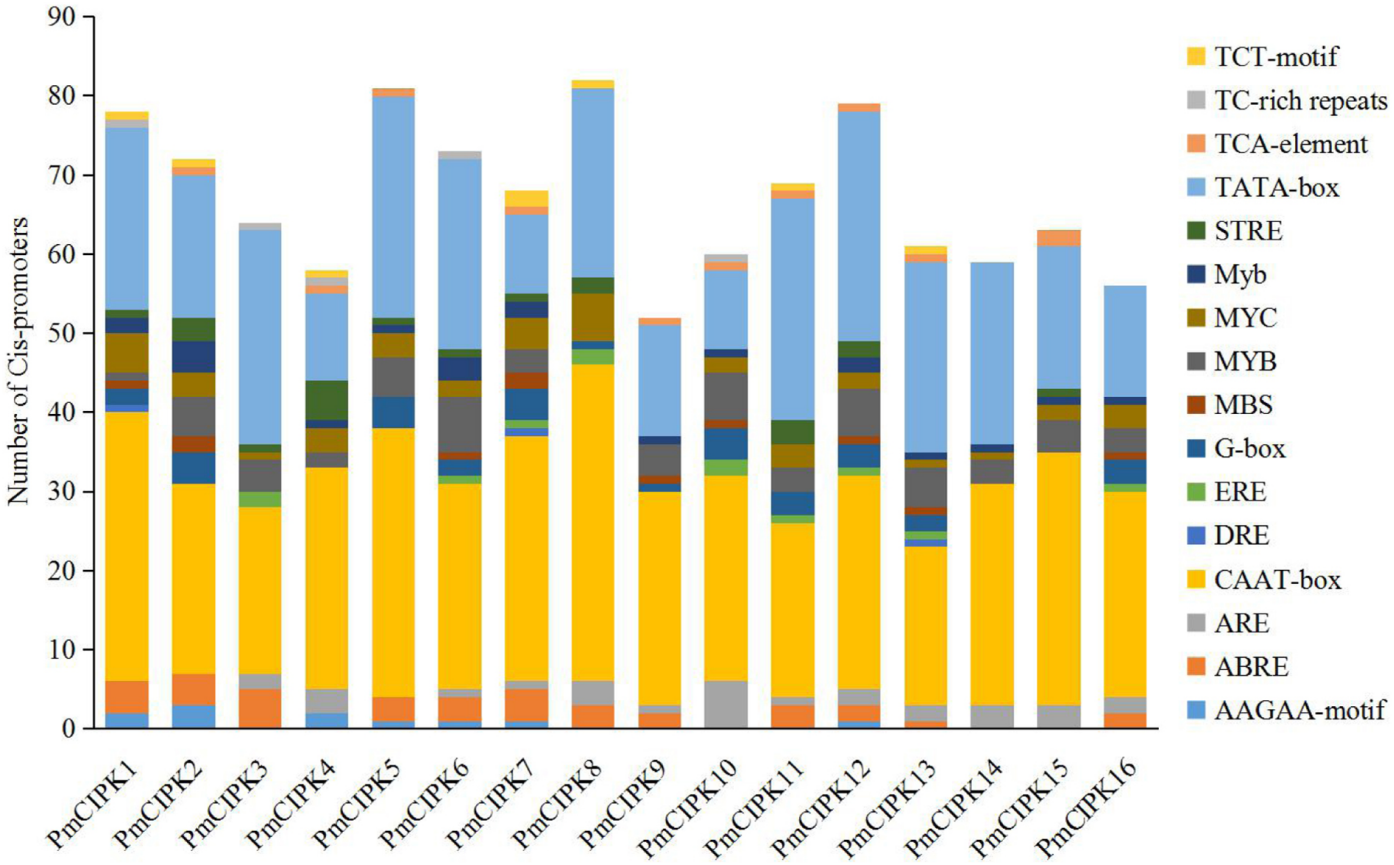
Types and number of cis-promoters involved in the stress response. The *x*-axis represents 1.5 kb upstream promoter region of *PmCIPK* genes. The *y*-axis represents number of cis-promoters.

The structure of a promoter affects the affinity of the promoter and RNA polymerase, thereby affecting the level of gene expression. Cis-acting regulatory elements related stress responses, including MYB, MYC, DRE, ABRE, and TC-rich repeats were identified in the promoter of *PmCIPK*s (Fig. 3). These elements were unevenly distributed in the promoter regions and the number of MYBs was relatively abundant. MYBs are considered crucial TFs in response to water, salt, drought, and cold (Li, Ng & Fan, 2015; Urao et al., 1993). Most of the *PmCIPKs*, except *PmCIPK4*, *PmCIPK10*, *PmCIPK14*, and *PmCIPK15*, contained ABRE (ACGTG) cis-promoter elements. ABRE is involved in abscisic acid (ABA) and VP1 responsiveness to abiotic stresses (Hattori, Terada & Hamasuna, 1995; Hobo et al., 1999). Meanwhile, DRE and TC-rich repeats were found in *PmCIPK* promoter regions, which respond to dehydration and cold stress or high-salt stress (Germain et al., 2012; Narusaka et al., 2003).

**Figure 4:**
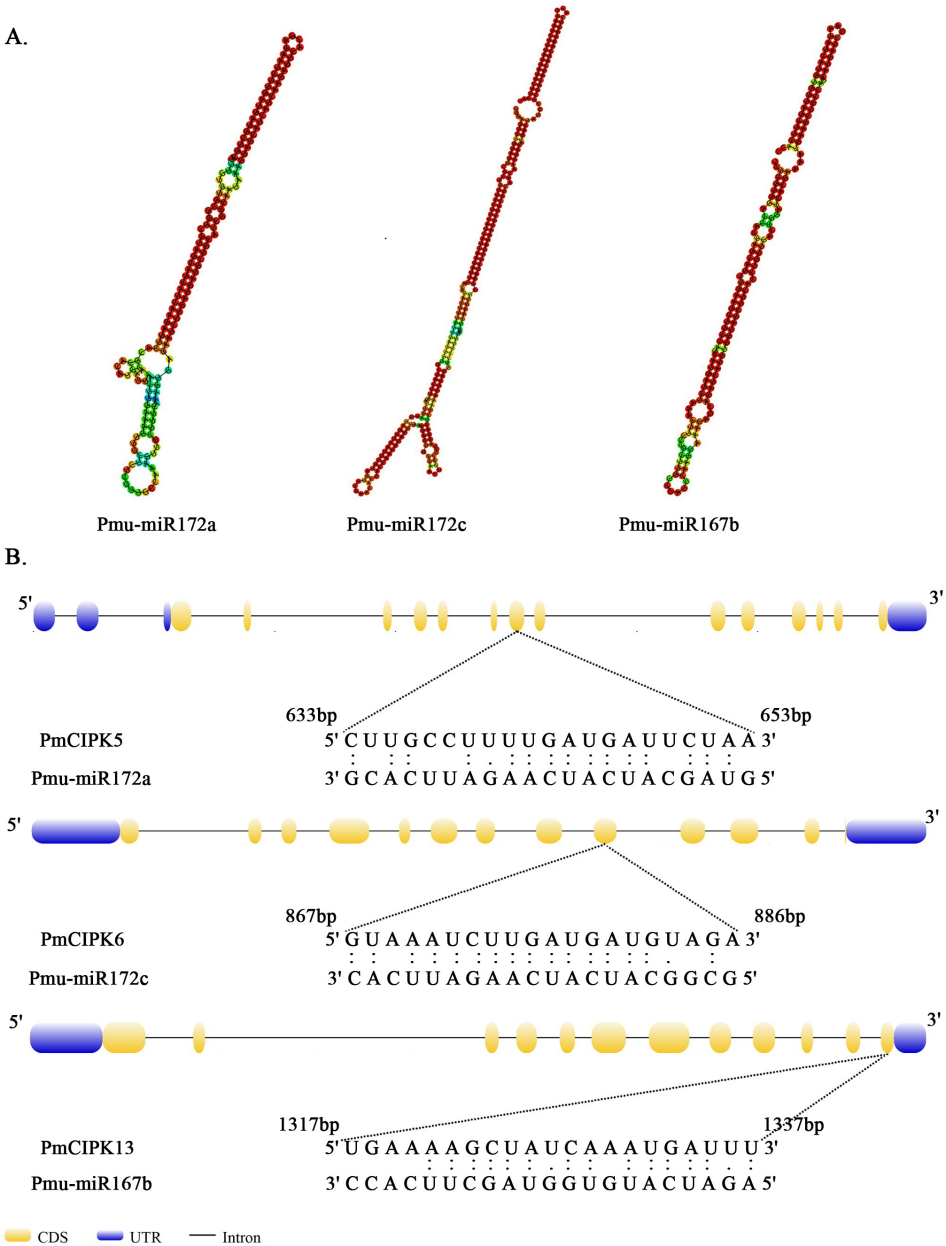
Analysis of mature Pmu-miRNA sequences and their corresponding target sites. (A) Secondary structures of Pmu-miR172a, Pmu-miR172c, and Pmu-miR167b precursor sequences. (B) Map of Pmu-miR172a, Pmu-miR172c, and Pmu-miR167b target sites.

MicroRNAs are important regulators of gene expression associated with abiotic stress in plants. Recent studies showed that miR167 and miR172 play a key role in the low temperature stress response in plants (Koc, Filiz & Tombuloglu, 2015; Zhang et al., 2008). In *P. mume*, we found that four miR172 (Pmu-miR172a-d) and four miR167 (Pmu-miR167a-d) members had been identified (Wang et al., 2014). Notably, two *PmCIPKs* (*PmCIPK5* and *PmCIPK6*) were targeted by Pmu-miR172s, and only *PmCIPK13* was targeted by Pmu-miR167s in the psRNATarget online software (Fig. 4). We drew the energy dot plot for Pmu-miR172a, Pmu-miR172c, and Pmu-miR167b, and the δ*G* values were 3.7, 5.9, and 3.9 kcal/mol at 37 °C in the plot file, respectively (Fig. S5). The energy required for the microRNA target is considered an important determinant of the response of mRNAs to miRNAs (Kertesz et al., 2007). miR167 and miR172 are highly conserved miRNA family members among plants (Jones-Rhoades & Bartel, 2004). Wang et al. (2014) have shown that Pmu-miR167 and Pmu-miR172 family members negatively regulate their targets during the flower bud development process.

### Expression pattern analysis of *PmCIPKs*

**Figure 5:**
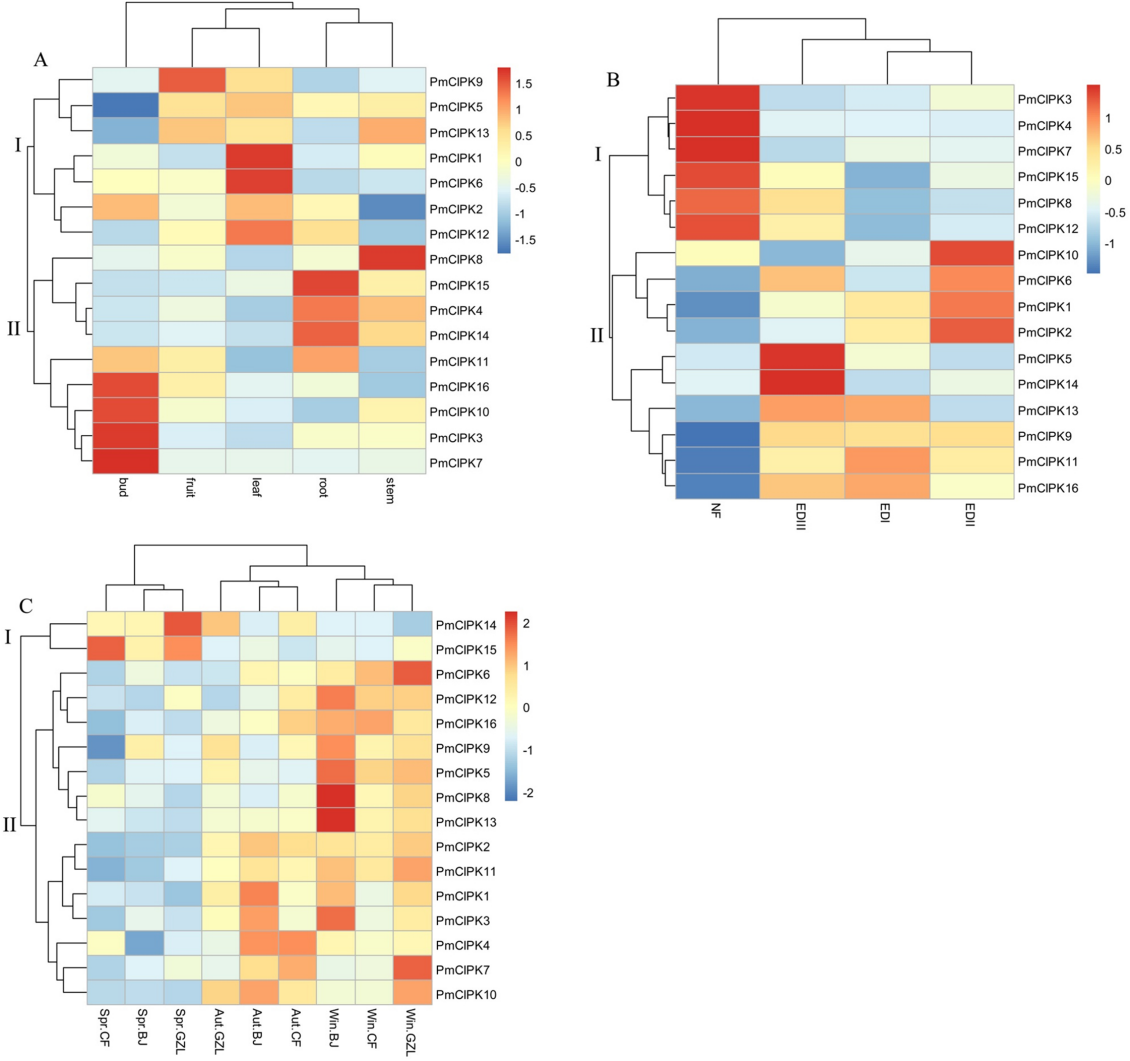
Expression profiles of *PmCIPK* genes under different conditions. (A) Expression profiles of *PmCIPKs* in different tissues. (B) Expression profiles of *PmCIPKs* in the flower bud during dormancy release. EDI, November; EDII, December; EDIII, January; NF, February. (C) Expression profiles of *PmCIPKs* in stems in different seasons and regions. Aut, Autumn; Win, Winter; Spr, Spring. BJ, Beijing; CF, Chifeng; GZL, Gongzhuling.

In different tissues (i.e., bud, leaf, root, stem, fruit), 16 *PmCIPK* gene heat maps showed differential expression, suggesting specific functions at particular stages of development (Fig. 5A; Table S5). For instance, seven genes (*PmCIPK1*, *PmCIPK2*, *PmCIPK5*, *PmCIPK6*, *PmCIPK9*, *PmCIPK12*, and *PmCIPK13*) exhibited high expression in the leaf. All of the *PmCIPK* genes were expressed during the bud dormancy process, and most of them had specific functions at particular stages of development (Fig. 5B; Table S6). We found that 6 *PmCIPKs* in subset I showed high expression levels at the NF stage, whereas subset II genes were significantly down-regulated except for *PmCIPK10*. Some of the genes were significantly up-regulated during the dormancy-breaking process. For example, four *PmCIPK* genes (i.e., *PmCIPK1*, *PmCIPK2*, *PmCIPK6*, and *PmCIPK10*) were simultaneously up-regulated at the EDII stage, while others, including *PmCIPK5* and *PmCIPK14,* were induced at the EDIII stage.

To further analyse the expression patterns of *PmCIPK* genes under cold response, *PmCIPK* genes were clustered based on different growth regions and periods. We found that the expression profiles of *PmCIPK* genes were significantly clustered in three different periods (autumn, winter, and spring). The expression levels of most *PmCIPK* genes was significantly up-regulated in autumn and winter, showing a response to cold acclimation and low-temperature stress. However, *PmCIPK14* and *PmCIPK15* showed high expression levels in the spring. According to the clustering analysis of *PmCIPK* genes in different regions, the results are similar to those obtained at different periods (Fig. 5C; Table S7). Most up-regulated *PmCIPK* genes were clustered in winter. The clustering results showed that the expression levels of most *PmCIPK* genes was up-regulated at low temperature. Among the up-regulated genes, we identified a *SnRK* (*SNF1-related protein kinase*, *Pm004487*) that could respond to the ABA complex and MAPK pathways of stress signalling in plants.

**Figure 6:**
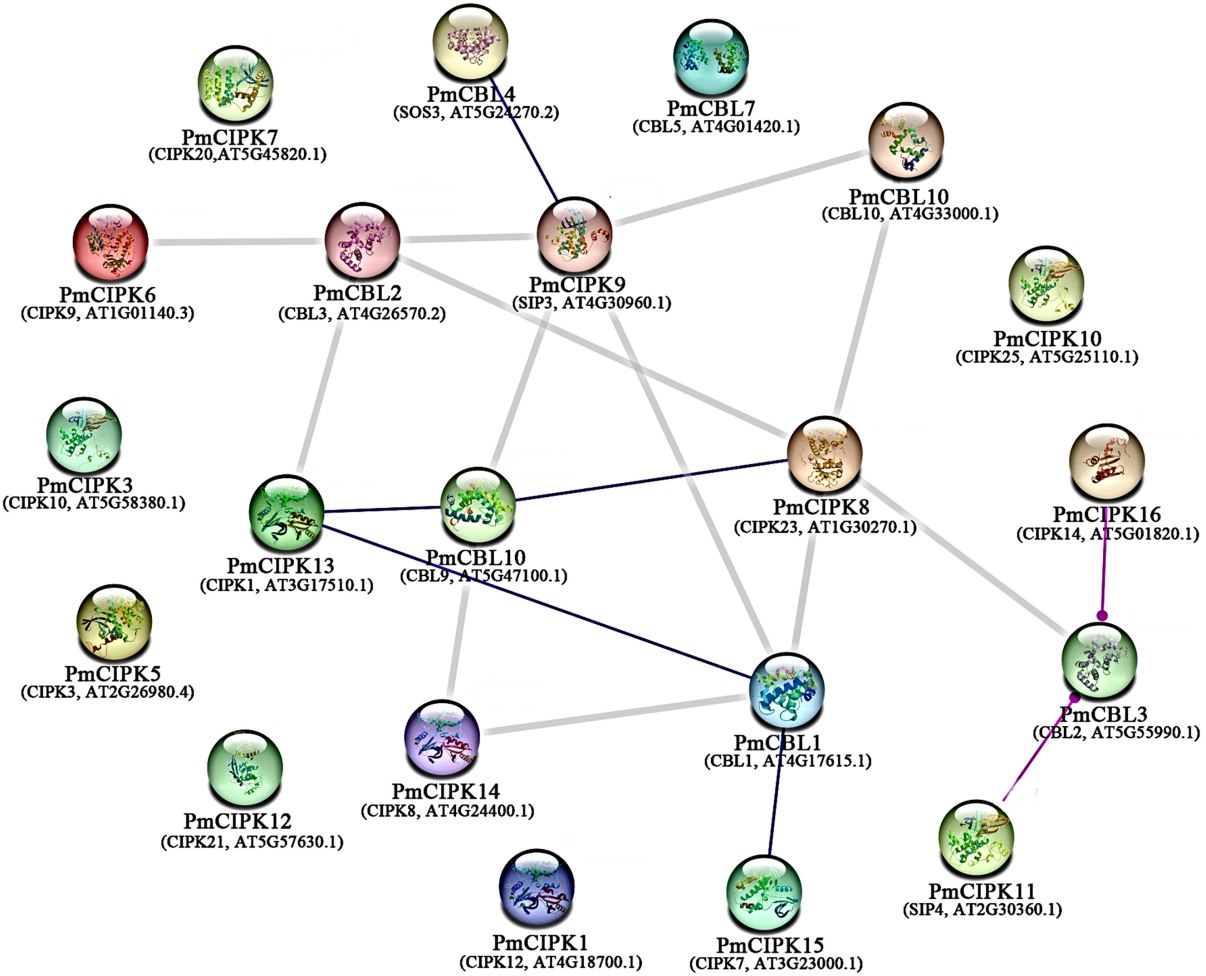
Interaction networks of PmCIPKs based on *A. thaliana* orthologues in the STRING database. Minimum required interaction score:highest confience (0.900).

To examine the signalling pathways among PmCIPK proteins, KOALA (KEGG Orthology And Links Annotation) analysis based on the K number assignment of KEGG GENES using SSEARCH computation was carried out. The PmCIPK13 sequence was identified in the scoring scheme for K number, and the K number was K07198. The K07198 number is associated with the AMP-activated protein kinase (AMPK) signalling pathway (ko04152). The major stress signalling pathways in plants are associated with the mammalian AMPK kinase, suggesting that these pathways may have evolved from an energy-aware pathway (Zhu, 2016). The catalytic domain of PmCIPK13 was similar to that of the mammalian AMPK. To further elucidate the putative function of PmCIPKs, the predicted interactions of PmCIPKs were established based on homologous proteins in *A. thaliana* (Fig. 6). The interaction network showed there is no direct interaction between the CIPKs, but the interaction network links between the CIPKs through CBLs interaction and has been reported in many species. PmCIPK13 may play an important role in the interaction network, which are associated with abiotic stress tolerance.

### Quantification of gene expression

**Figure 7:**
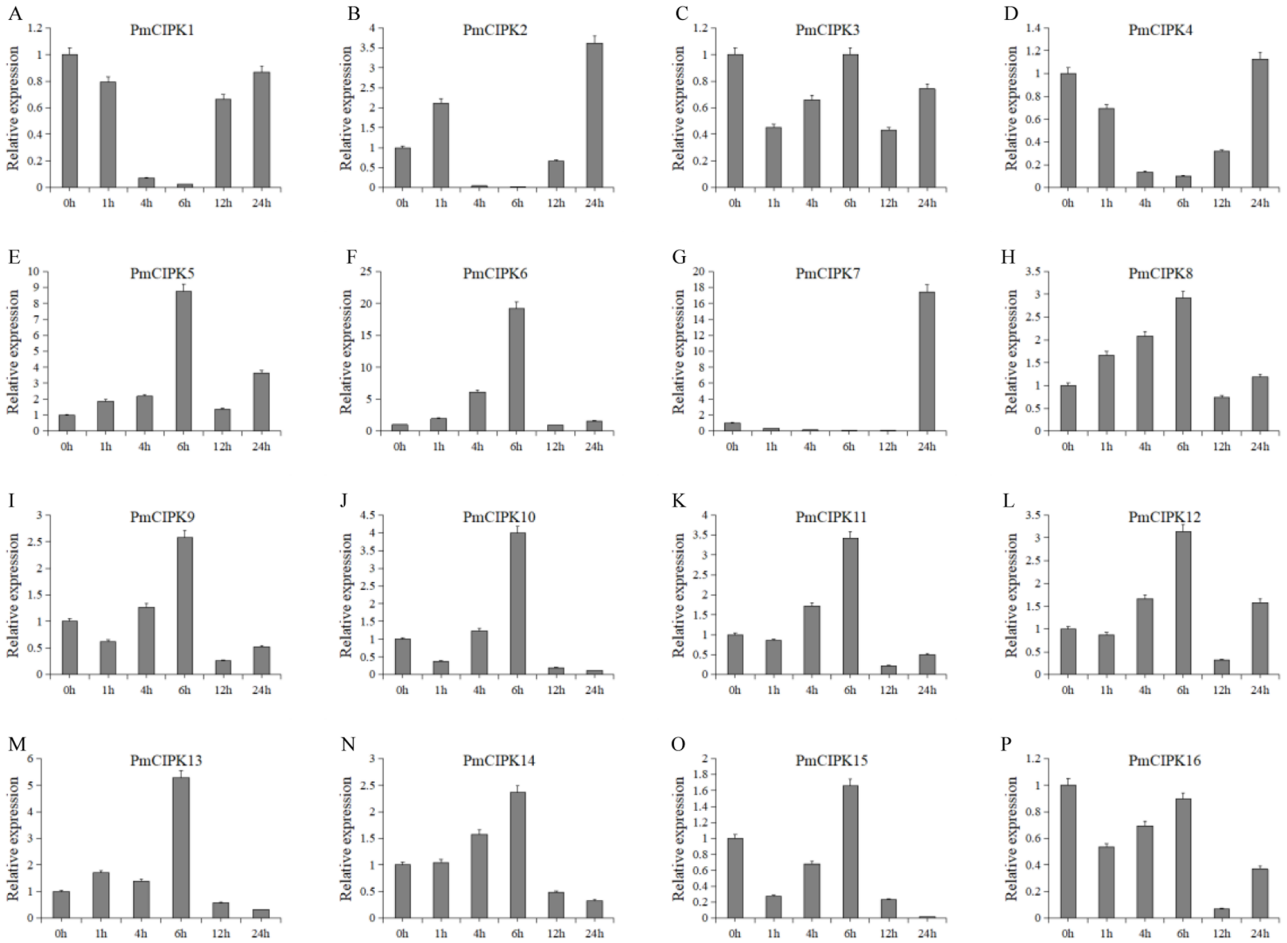
Expression level of *PmCIPK* genes under 4 °C treatment. The expression level of the 16 *PmCIPK* genes (A–P) were gained by qRT-PCR. *Protein Phosphatase 2A* (*PP2A*) gene was used as the internal control to standardize for each reaction.

Cold acclimation was found to contribute to the maximum freezing tolerance of plants. To investigate the function of *PmCIPKs*, the expression levels of *PmCIPKs* under low temperature treatment were examined by qRT-PCR experiments. The *PmCIPK* genes were up-regulated or down-regulated in the time course expression levels during 4 °C treatment (Fig. 7). We have compared the gene expression patterns of these *CIPK* genes across *P. mume*, *A. thaliana,* and *O. sativa* during cold stress and some *CIPK* genes expression levels are similar in the plants (Fig. S6). The majority of *PmCIPKs* were up-regulated within 4 or 6 h except for *PmCIPK1*, *PmCIPK2*, *PmCIPK4*, and *PmCIPK7*, and the highest expression levels were found at 6 h before they were down-regulated with the prolongation of cold treatment time. We found that the target genes of Pmu-miR172/167 (*PmCIPK5, PmCIPK6, PmCIPK13*) were up-regulated following cold treatment compared to those of the control while the expression levels of other genes (*PmCIPK3, PmCIPK16*) were relatively stable. Only *PmCIPK7* was rapidly up-regulated at 24 h under 4 °C treatment. The expression levels of *PmCIPK5, PmCIPK6, PmCIPK8,* and *PmCIPK13* were up-regulated and had similar changes as the *PmCBF* gene that has been proven to be related to cold response (Zhang et al., 2013b).

## Discussion

Freeze stress hinders the growth of most plants and becomes an increasing threat to the expanded cultivation of plants such as the perennial woody plant *P. mume*. Wheat crops grown under normal growth temperature conditions are killed by freezing temperatures at approximately −5 °C, but the species can survive temperatures as low as −20 °C after cold acclimation (Thomashow, 1999). As reported in previous research, Ca^2+^ plays an important role in the cold stress response. The functions of the CIPK-CBL complex are closely related to Ca^2+^. The expression levels of *CBF/DREB1* genes were enhanced by a vacuolar Ca^2+^/H^+^ antiporter (CAX1) to exhibit increased freezing tolerance (Catala et al., 2003). The cold stress-related genes were cloned, functional assays using the complete genome were carried out, and the exact roles of C-repeat/DRE binding factor (CBF/DRE) about cold tolerance have been investigated in *P. mume* (Sun et al., 2015; Zhang et al., 2013b). The expression levels of *PmCIPKs* were up-regulated or down-regulated by cold treatment, however, there are differences in the expression levels of these genes (Fig. 7). These expression levels were similar to those of their homologs in wheat and maize (Chen et al., 2011; Sun et al., 2015), indicating that *PmCIPKs* might play important roles in cold response.

Domestication has been a conventional breeding method for crop improvement, however, improvements to create useful traits, including those of *P. mume,* have required more time via conventional breeding. Plants have evolved during long-term breeding. The percentage of shared genes is as high as 55.0% among six Rosaceae species (*P. mume, P. persica*, *F. vesca*, *P. avium*, *M. × domestica*, and *P. yedoensis*), as shown by genome comparative analysis involving TFs, functional genes and uncharacterized genes (Chagné et al., 2014). The *P. mume* genome contains nine ancestral chromosomes, which is unique in the Rosaceae family, revealing that the ancestral chromosomes evolved into eight current chromosomes in *P. mume* after 11 fusion events, seven current chromosomes in the strawberry after 15 fusion events, and 17 current chromosomes in the apple after one whole genome copy and five fusion events (Shulaev et al., 2011; Velasco et al., 2010; Zhang et al., 2012). We identified a range of 16–30 *CIPK* genes each from 10 Rosaceae species, whereas, the number of *CIPK* genes in *Prunus* species is relatively concentrated, ranging from 16 to 20. Among studies on the phylogenetic tree of PmCIPK, *Prunus* species showed closer affinity relationships in the Rosaceae family (Fig. 1), and genome resequencing analysis indicated a strong signature of introgressions in *Prunus* species (Zhang et al., 2018a). The diversification of *Prunus* genomes traces back to pre-66 Mya, and then a successive split period (36–44 Mya) arose (Chagné et al., 2014).

The NAF domain is found in a plant-specific subgroup of CIPKs that mediate the interaction with the CBL calcium sensor proteins. The CIPK-CBL complexes may connect low temperature-induced calcium signalling with the ICE or SnRK2.6/OST1 cascades, because CIPK-CBL binds to calcium and calmodulin (Zhu, 2016). Recent studies have shown that low temperature can activate SnRK2.6/OST1 and that SnRK2.6 interacts with and phosphorylates ICE1 to regulate the CBF-COR gene expression during cold stress induction and freezing tolerance (Ding et al., 2015). A total of 16 *PmCIPK* genes were identified based on the NAF domain, and most *PmCIPK* genes had significant transcript accumulation in the low temperature period. Among the up-regulated genes, we identified a *SnRK* (*SNF1-related protein kinase*, *Pm004487*) that could respond to the ABA complex and MAPK pathways of stress signalling in plants. Hormone signal transduction and MAPK signalling pathways play a critical role in abiotic stress in plants (Danquah et al., 2014). The binding of a CBL protein to the regulatory NAF domain of a CIPK protein causes the activation of the kinase in a calcium-dependent pattern (Albrecht et al., 2001). The CBL/CIPK complex acts as a Ca^2+^ sensor involved in ABA signalling and stress-induced ABA biosynthesis pathways and contributes to the regulation of early stress-related CBF/DREB TFs (Guo et al., 2002).

Tissue-specific transcription profiles of *PmCIPK* genes were identified and most of the *CIPK* genes were differentially expressed in different tissues and at different stages of dormancy release. *PmCIPK*s were expressed specifically in the leaf and stem, and had similar tissue-specific expression profiles to *CIPKs* from *M. × domestica* and *O. sativa* (Kanwar et al., 2014; Niu et al., 2018). *PmCIPK4*, *PmCIPK7*, *PmCIPK10*, and *PmCIPK11* were expressed in buds whereas *PmCIPK5*, *PmCIPK9*, and *PmCIPK13* were expressed in fruits, suggesting that they might affect seed size and embryonic development, which is similar to *AtCIPKs* in *A. thaliana* (Eckert et al., 2014). Notably, *PmCIPK3*, *PmCIPK7*, *PmCIPK8*, *PmCIPK12*, *PmCIPK15*, and *PmCIPK16* were expressed at low levels during the dormancy stages and quickly up-regulated during the dormancy release stage. We speculated that these *PmCIPKs* might play important roles in the processes of dormancy release. Expression of *AtCBL1* is induced by cold stress and AtCBL1 associates with AtCIPK7, which mediates plant responses to cold stress (Huang et al., 2011). Similarly, *BdCIPK31* improved osmoprotectant biosynthesis and ROS detoxification to enhance low temperature tolerance in transgenic tobacco (Luo et al., 2018). Here, *PmCIPK1*, *PmCIPK2*, *PmCIPK5*, *PmCIPK6*, *PmCIPK10*, *PmCIPK13*, and *PmCIPK14* were significantly regulated in the depths of winter (Fig. 5) and might have been involved in the low temperature response to protect flower buds at subfreezing temperatures.

When plants encounter cold stress, a series of cellular activities and molecular mechanisms can be activated that permit the plant to adapt to low temperatures (Shi, Ding & Yang, 2018). Previous studies have shown that miR167 and miR172 play key roles in the cold stress response in plants (Koc, Filiz & Tombuloglu, 2015; Zhang et al., 2008). In *P. mume*, two *PmCIPKs* (*PmCIPK5* and *PmCIPK6*) were targeted by Pmu-miR172s, and only *PmCIPK13*, which belongs to Group I, was targeted by Pmu-miR167s (Fig. 1; Fig. S2). These genes contained multiple exons, and their target sites were all located in the coding region (Fig. 4). *PmCIPK5* is highly homologous to the *Arabidopsis AtCIPK3* (*AT2G26980.4*) gene. The expression of *AtCIPK3* is responsive to cold, drought, ABA, high salt, and wounds, and *AtCIPK3* can be used as a “node” between the ABA-dependent/ABA-independent pathways in the cold response signalling pathway (Kim, 2003). Xiang, Huang & Xiong (2007) reported that the overexpression of the *OsCIPK3* gene could dramatically strengthen tolerance to cold stress in transgenic plants. These proteins included a putative protein phosphatase 2C (PP2C) binding site (Vlad et al., 2009). The core ABA co-receptor complex, including the PP2C protein, plays an essential role in the response to various adaptive stresses (Guo et al., 2001). Three cold response genes (*PmCIPK5*, *PmCIPK6*, and *PmCIPK13*) were screened out, which laid a foundation for subsequent studies. However, there are still many gaps in our knowledge of the cold response mechanisms underlying cold acclimation processes, and further analyses are needed to increase our understanding of cold acclimation. This is not only essential for defining a molecular mechanism for the cold acclimation processes but also has important implications for agricultural production in geographical distributions where crop and horticultural plant species can be planted.

## Conclusions

Although the molecular functions of some CIPK-CBL protein complexes have been verified in herbaceous plants *A. thaliana* and *O. sativa*, their functions in woody plants are still unclear. In this study, we performed the first genome-wide identification of the *CIPK* gene family in *P. mume*. Sixteen *PmCIPK* genes were identified, and 12 *PmCIPK* genes including Pmu-miR172/167-targeted genes (*PmCIPK5*, and *PmCIPK6*) were up-regulated by cold treatment. Nine *PmCIPK*s were highly expressed in flower buds in December and January, and twelve *PmCIPK*s were up-regulated in stems in winter. Our results suggest that the roles of *PmCIPKs* in regulating the stress response to low temperature may supply freezing tolerance to plants, especially in enduring the freezing period of winter weather.

## Supplemental Information

10.7717/peerj.6847/supp-1 Supplemental Information 1 Chromosomal locations of *PmCIPK* genes on choromosomes.

10.7717/peerj.6847/supp-2 Supplemental Information 2 Phylogenetic tree of CIPK sequences from *P. mume*, *A. thaliana*, and *O. sativa*.

10.7717/peerj.6847/supp-3 Supplemental Information 3 Schematic diagram of PmCIPK protein motifs.

10.7717/peerj.6847/supp-4 Supplemental Information 4 Predicted tertiary structures of PmCIPK proteins.

10.7717/peerj.6847/supp-5 Supplemental Information 5 The energy dot plot for Pmu-miR172a, Pmu-miR172c, and Pmu-miR167b.

10.7717/peerj.6847/supp-6 Supplemental Information 6 Expression patterns of *CIPK* genes during cold stress.

10.7717/peerj.6847/supp-7 Supplemental Information 7 PmCIPK genes of forward and reverse primers.

10.7717/peerj.6847/supp-8 Supplemental Information 8 Information for the proteins used in the present study.

10.7717/peerj.6847/supp-9 Supplemental Information 9 Divergence between *CIPK* gene pairs in *P. mume*.

10.7717/peerj.6847/supp-10 Supplemental Information 10 The information of homologous relationship of PmCIPKs.

10.7717/peerj.6847/supp-11 Supplemental Information 11 Expression profiles of PmCIPK genes in different tissues.

10.7717/peerj.6847/supp-12 Supplemental Information 12 Expression profiles of PmCIPK genes during the process of flower bud dormancy release.

10.7717/peerj.6847/supp-13 Supplemental Information 13 Expression profiles of *PmCIPK* genes in different regions and seasons.

## Additional Information and Declarations

### Data Availability

The following information was supplied regarding data availability: Raw data are available as Supplemental Files.
