# Supplementary figures and images for: Identification and comparative analysis of the CIPK gene family and characterization of the cold stress response in the woody plant Prunus mume
Source: PeerJ. 2019 Apr 30;7:e6847. doi: 10.7717/peerj.6847 (PMC6499057; doi:10.7717/peerj.6847)

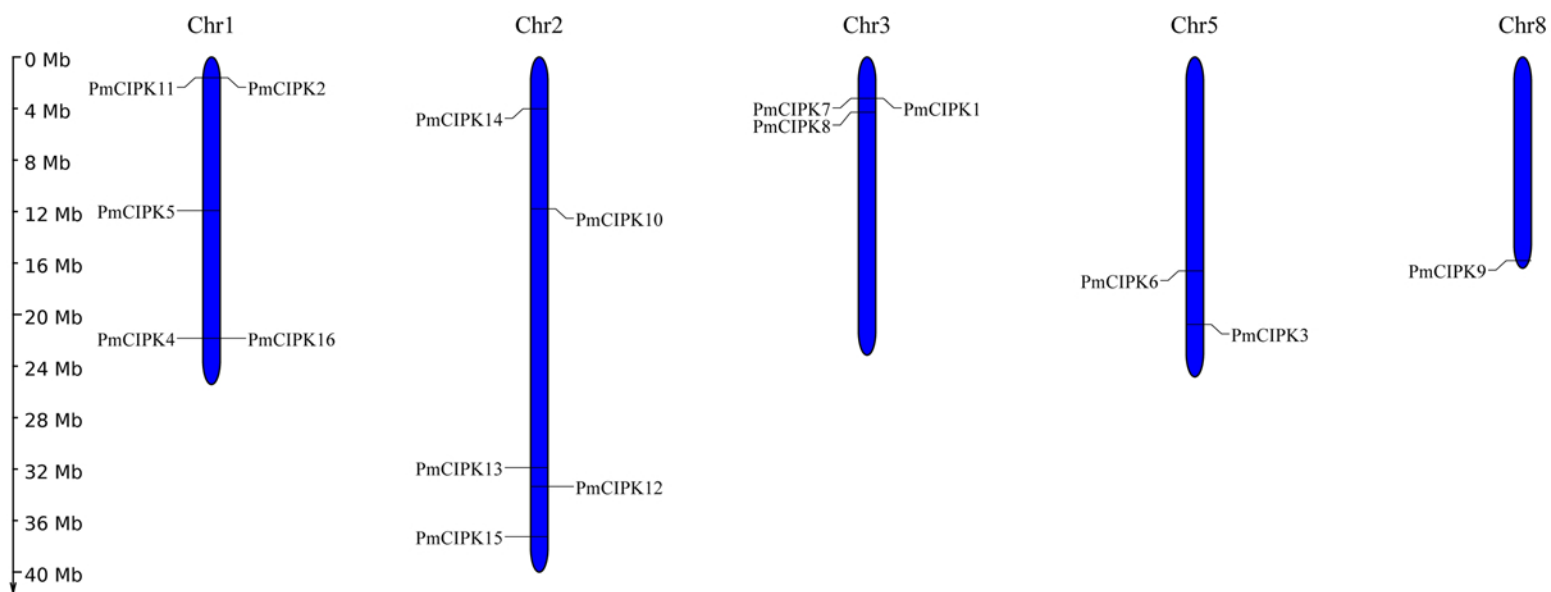

Supplemental Figure S1. Chromosomal locations of *PmCIPK* genes on chromosomes

Supplement: Supplemental Information 1 [file peerj-07-6847-s001.pdf]

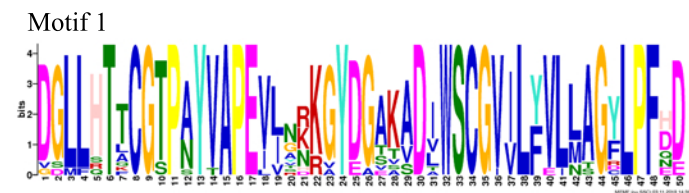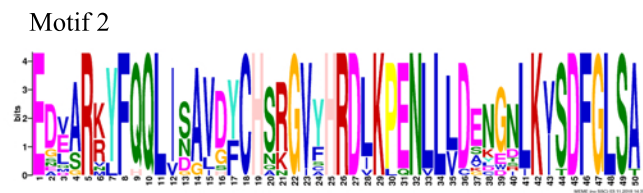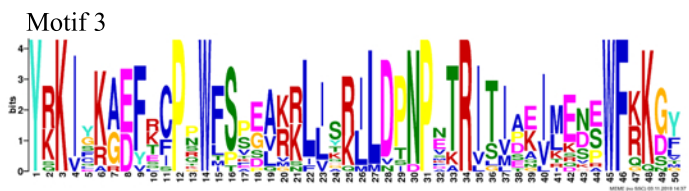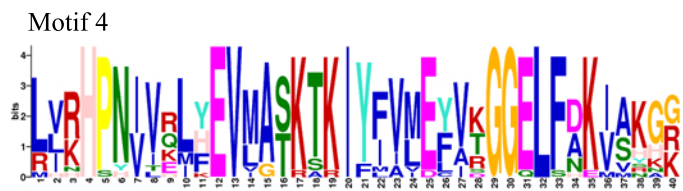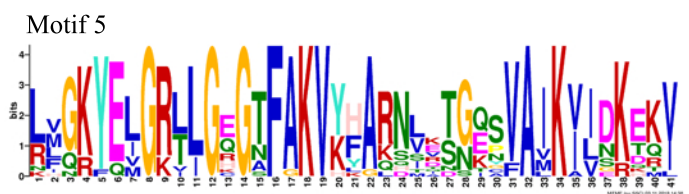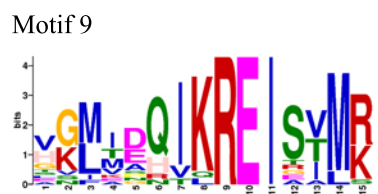

Supplementary Figure S3. Schematic diagram of PmCIPK protein motifs

Supplement: Supplemental Information 3 [file peerj-07-6847-s003.pdf]

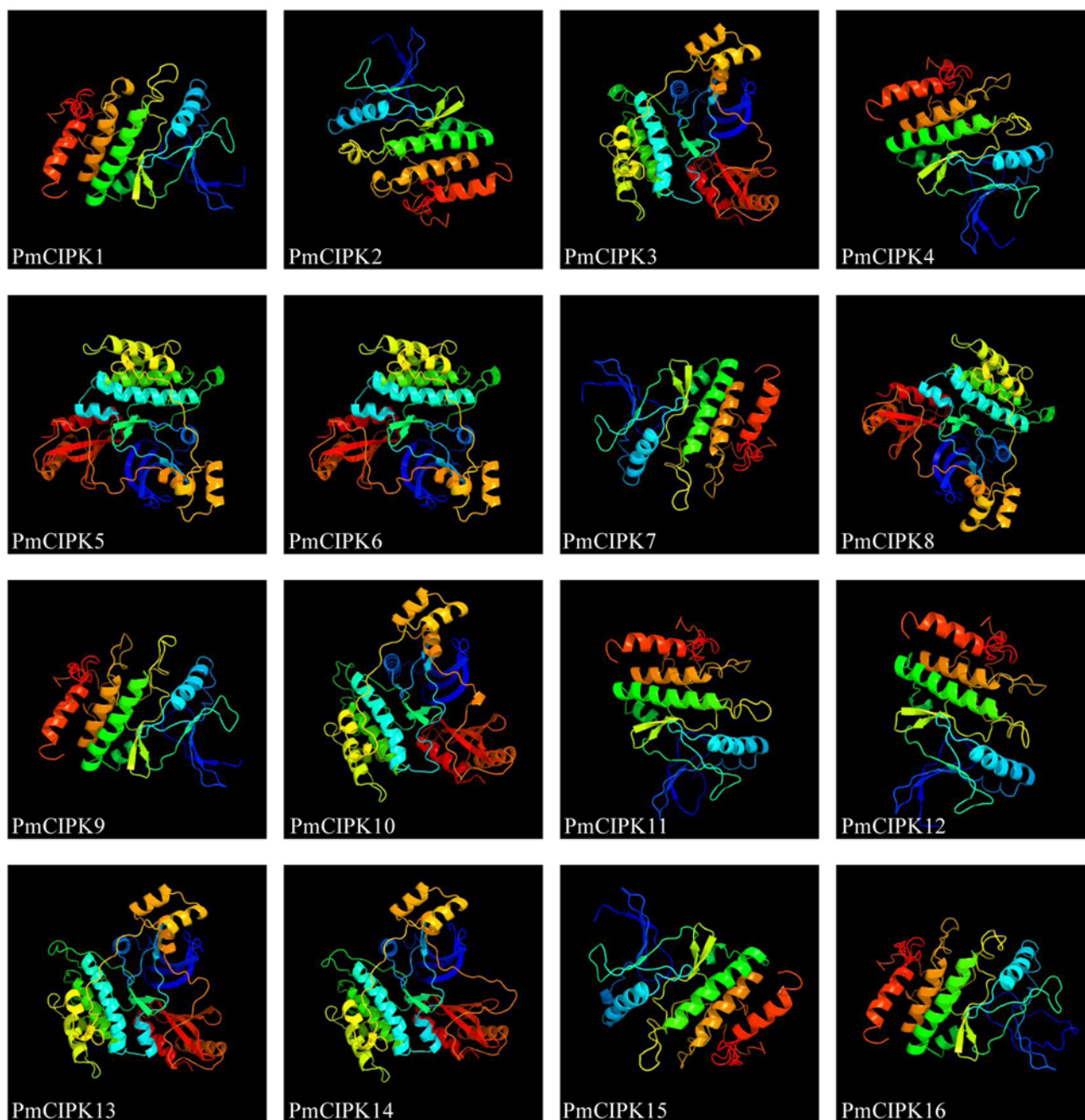

Supplementary Figure S4. Predicted tertiary structures of PmCIPK proteins

Supplement: Supplemental Information 4 [file peerj-07-6847-s004.pdf]

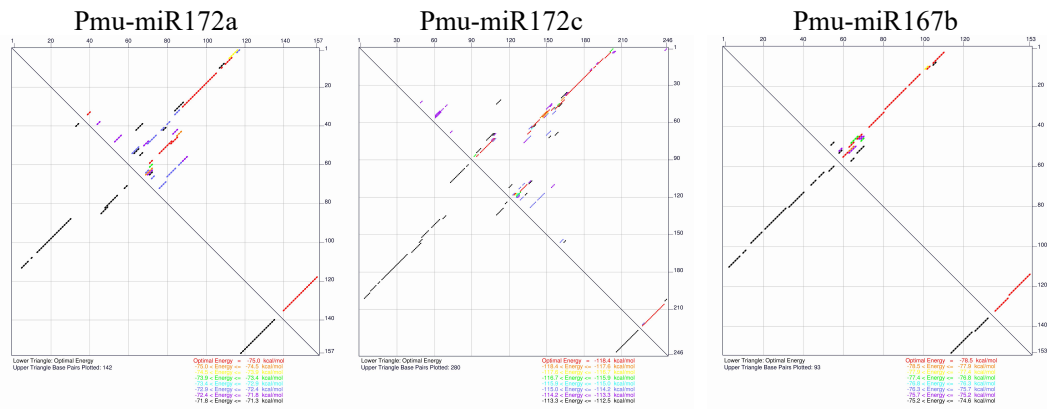

Supplementary Figure S5. The energy dot plot for Pmu-miR172a, Pmu-miR172c, and Pmu-miR167b

Supplement: Supplemental Information 5 [file peerj-07-6847-s005.pdf]
