# Supplementary material for: Identification and comparative analysis of the CIPK gene family and characterization of the cold stress response in the woody plant Prunus mume
Source: PeerJ. 2019 Apr 30;7:e6847. doi: 10.7717/peerj.6847 (PMC6499057; doi:10.7717/peerj.6847)
Supplement: Supplemental Information 2 [file peerj-07-6847-s002.pdf]

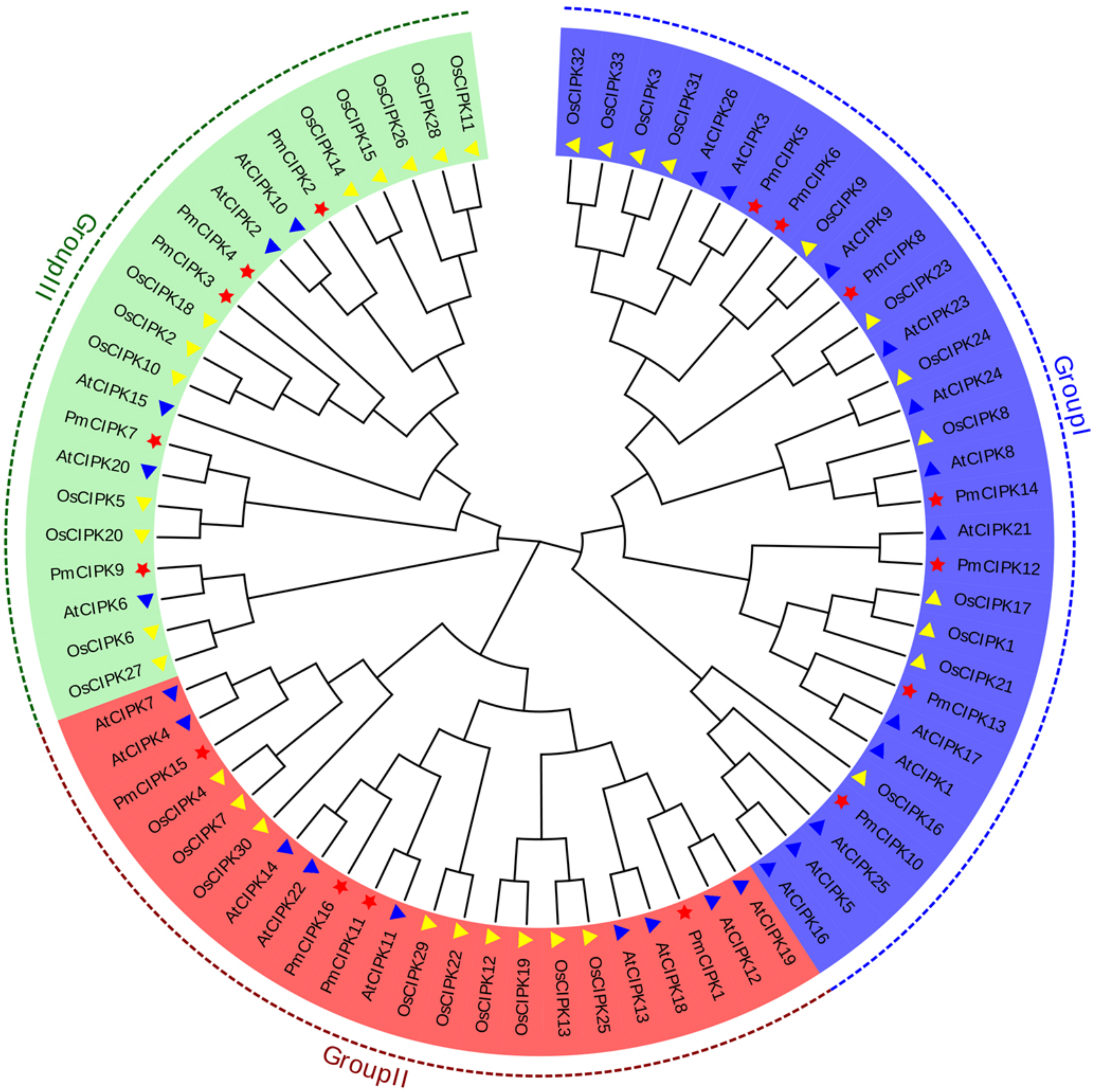

Supplementary Figure S2. Phylogenetic tree of CIPK sequences from *P. mume*, *A. thaliana*, and *O. sativa*. The subfamilies Group I, Group II, and Group III are indicated by blue, red, and green branch, respectively. Pm, *P. mume*; At, *A. thaliana*; and Os, *O. sativa*.
