# Supplementary material for: Identification and comparative analysis of the CIPK gene family and characterization of the cold stress response in the woody plant Prunus mume
Source: PeerJ. 2019 Apr 30;7:e6847. doi: 10.7717/peerj.6847 (PMC6499057; doi:10.7717/peerj.6847)
Supplement: Supplemental Information 6 [file peerj-07-6847-s006.pdf]

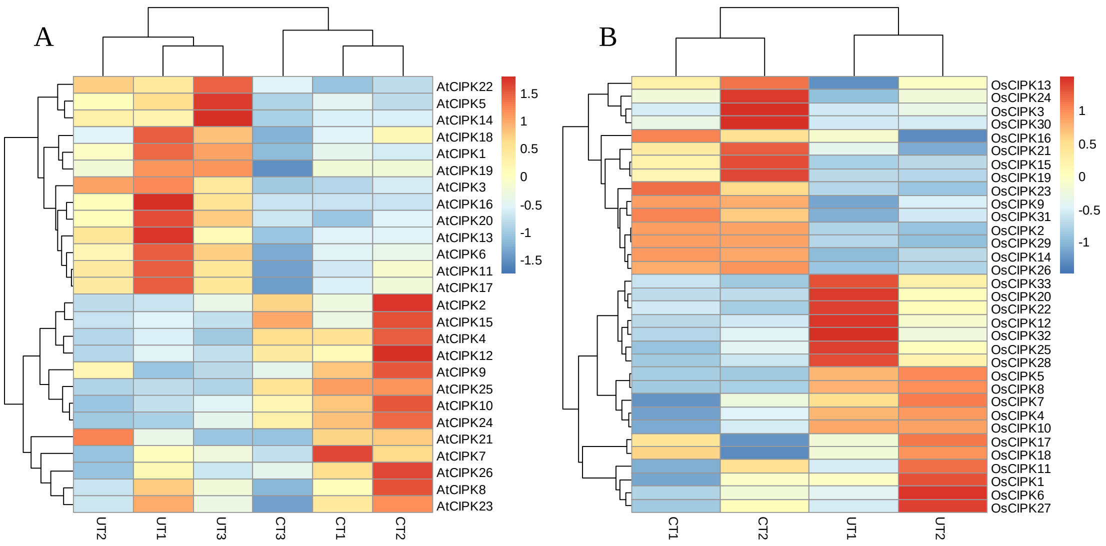

Supplemental Figure S6. Expression patterns of *CIPK* genes during cold stress. A, Expression patterns of *CIPK* genes in *A. thaliana*. B, Expression profiles of *CIPK* genes in *O. sativa*. CT, Chilling treatment; UT, Unchilling treatment.
